# Full-ocean-depth EM124 mapping of the Challenger Deep by R/V Hakuho-maru and depth-estimate sensitivity tests

**DOI:** 10.1038/s41597-026-08267-z

**Published:** 2026-09-16

**Authors:** Masakazu Fujii, Chiori Tamura, Yasuhiko Ohara, Kyoko Okino

**Affiliations:** 1 National Institute of Polar Research, Tachikawa 190-8518, Japan; 2 SOKENDAI (The Graduate University for Advanced Studies), Hayama 240-0193, Japan; 3 Atmosphere and Ocean Research Institute, The University of Tokyo, Kashiwa 277-8564, Japan; 4 Hydrographic and Oceanographic Department of Japan, Tokyo 100-8932, Japan; 5 Nagoya University, Nagoya, Japan; 6 Tokai University, Shizuoka 424-8610, Japan

## Abstract

The Challenger Deep in the Mariana Trench is the deepest known point on Earth, yet recent depth estimates still differ by several meters because of environmental and instrumental uncertainties. Here we present a new full-ocean-depth multibeam bathymetric dataset collected with a Kongsberg EM124 echosounder aboard R/V Hakuho-maru during the KH-23-9 cruise in 2023. The survey comprises east–west and north–south lines acquired at vessel speeds of 4–15 kt. Five seawater sound velocity models were constructed from contemporaneous XCTD data, historical full-depth CTD data, and a recent full-depth reference profile to test the sensitivity of depth estimates. After ping-by-ping editing and gridding, the preferred KH23XCTD+KH92CTD solution resolves three basins deeper than 10,900 m, with maximum depths of 10,926, 10,912, and 10,927 m in the western, central, and eastern basins, respectively. Alternative sound velocity models yield maximum depths from 10,914 to 10,932 m, illustrating the meter- to >10-m-scale sensitivity of full-ocean-depth multibeam estimates to the applied sound velocity structure. Cleaned point soundings, gridded bathymetric surfaces for each sound velocity model, and the gridding script are provided. The dataset enables assessment of how sound velocity structure, survey speed, and beam-footprint geometry influence depth estimates in full-ocean-depth multibeam mapping, and provides a new reference for cross-comparison of Challenger Deep bathymetry.

## Background & Summary

Exploration of the deepest regions of the Earth’s surface has been a longstanding scientific challenge spanning centuries. The Challenger Deep in the Mariana Trench (~11.3°N, 142.2°E) is recognized as the deepest part of the ocean floor. Acoustic bathymetric and pressure-based measurements in this region have a long and distinguished history, beginning with early soundings by the British ship HMS *Challenger II*^1^, followed by the Soviet ship *Vityaz* during the 1957 International Geophysical Year, and the American manned bathyscaph *Trieste* in 1960^2^.

Since these pioneering efforts, modern multibeam surveys have progressively improved our understanding of the trench geometry. Japanese vessels have played a key role, including S/V *Takuyo* in 1984^3^, R/V *Hakuho-maru* in 1992^4,5^, and R/V *Kairei* in 1998, 1999, and 2002^6,7^. Additional surveys by the American vessel USNS *Sumner* in 2010^8^, German ship R/V *Sonne* in 2016^9^, and DSSV Pressure Drop in 2019–2020^10,11^ have further refined the bathymetric and depth-control framework of the Challenger Deep.

Recent studies have also highlighted the difficulty of assigning a single absolute depth to the deepest point. Gardner *et al*.^8^ reported a 95% confidence uncertainty of ±25 m for their multibeam-derived estimate, while van Haren *et al*.^9^ reported an uncertainty of ±12 m based on multibeam bathymetry supported by full-depth CTD observations. The DSSV *Pressure Drop* survey, conducted with a Kongsberg EM124 multibeam echo sounder operating at 8 kt, yielded a deepest value of 10,924 ± 15 m in the eastern basin when combined with full-depth CTD profiles collected by the submersible DSV *Limiting Factor*^10^. Concurrently, Greenaway *et al*.^11^ reported a revised depth of 10,935 m ± 6 m at 95% confidence, derived from the submersible altimeter profiles referenced to *in-situ* pressure. Notably, Greenaway *et al*.^11^ incorporated detailed corrections for seawater density structure, atmospheric pressure, water level, local gravity anomalies, and vertical gravity gradients in converting pressure to depth. Such corrections are essential because even small deviations from standard gravity can accumulate into several meters of systematic bias at ~11 km depth.

Thus, although the Challenger Deep remains Earth’s deepest known location, small but persistent discrepancies among recent depth estimates highlight the need to document how survey conditions and processing choices influence full-ocean depth multibeam echo-sounding. In this study, we present new multibeam sounding data collected with the newly installed Kongsberg EM124 system aboard R/V Hakuho-maru during cruise KH-23-9 on 30 November 2023. Using these observations, we test the sensitivity of gridded depth estimates to seawater sound velocity structure, vessel speed, and system operating mode. In particular, differences among the sound velocity structure models considered here change the estimated maximum gridded depth by up to 18 m. We also examine how beam-footprint geometry and gridding choices affect the representation of small-scale depressions at full-ocean depth.

We further show that the resulting bathymetry, including the three principal basins that each exceed 10,900 m, is consistent with previous surveys in its large-scale morphology. The preferred KH23XCTD+KH92CTD solution gives a maximum gridded EM124 depth of 10,927 m in the eastern basin, whereas alternative sound velocity models yield maximum depths from 10,914 to 10,932 m. We therefore present the 10,927 m value as the preferred gridded solution for this dataset, not as a definitive redetermination of the deepest point on Earth. This dataset provides a basis for cross-comparison of Challenger Deep bathymetry and for evaluating how sound velocity structure, survey configuration, and processing choices influence full-ocean-depth multibeam mapping.

## Methods

### Multibeam echo sounding using EM124 installed on R/V Hakuho-maru

**Fig. 1:**
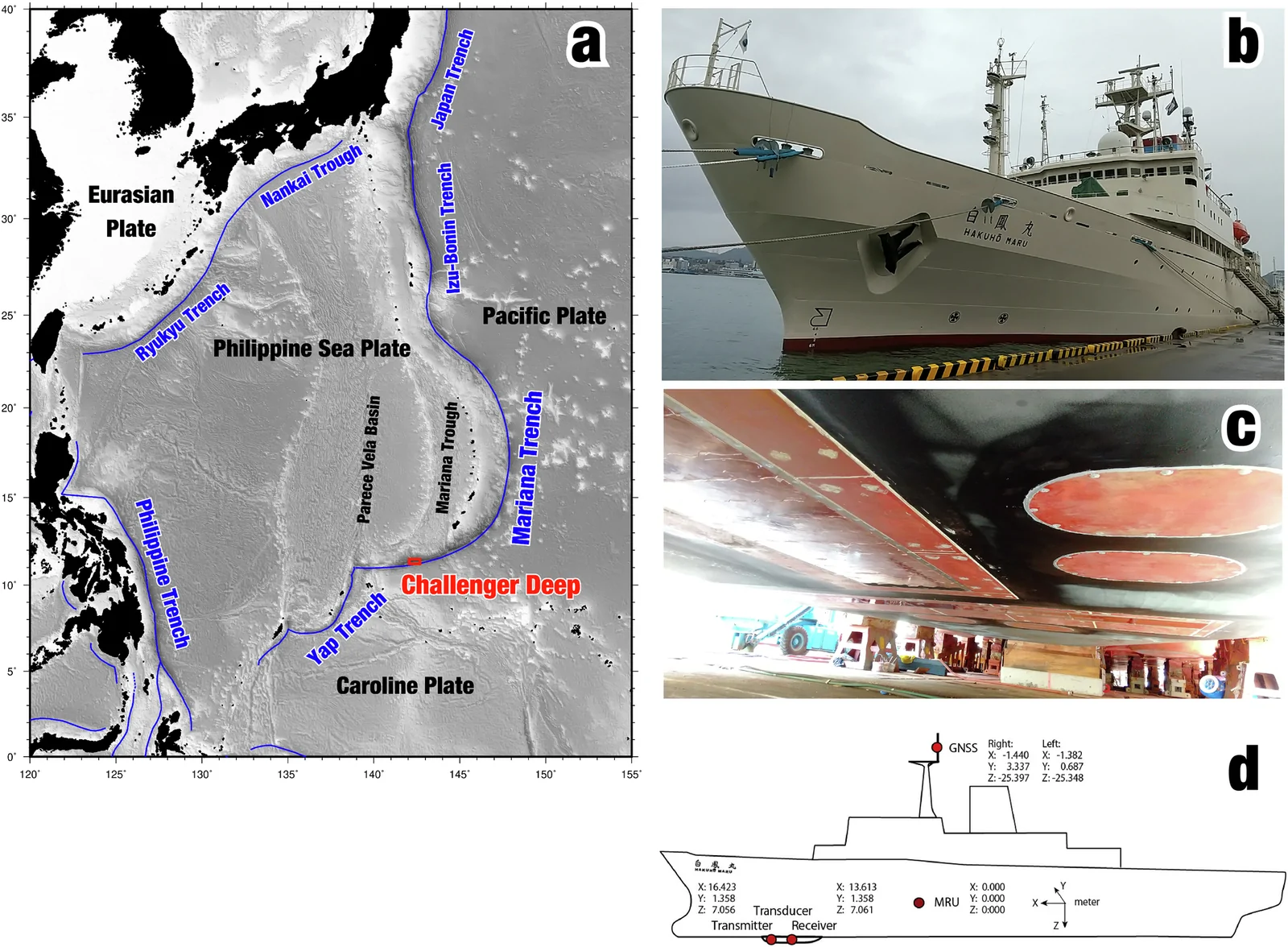
Bathymetric observation at the Challenger Deep during R/V Hakuho-maru cruise of KH-23-9. (**a**) survey area (red box) shown with trench geometry in plate subduction system surrounding Philippine Sea Plate. (**b**) overall view of R/V Hakuho-maru. (**c**) EM124 installed on the bottom of R/V Hakuho-maru. (**d**) instrument configuration of transducer, motion reference unit (MRU), and GNSS sensor.

Seafloor bathymetry was acquired using the Kongsberg EM124 multi-narrow-beam echosounder installed on R/V Hakuho-maru (Fig. 1). The system operates at sonar frequencies between 10.5–13.5 kHz, with a nominal frequency of 12 kHz. In deep-water modes, the transmit fan is divided into eight sectors. By employing the “Dual swath” mode, the system covers an angular sector of up to 150° and can form 1,024 beams per ping. The EM124 installation consists of acoustic transducer arrays, transmitter and receiver units (with a beamwidth of 2° by 2°), a processing unit, and a hydrographic workstation running SIS version 5.11.1 (built on 2022-11-11) on a Windows 10 computer. All acoustic data were recorded in .kmall format.

### KH-23-9 expedition

**Fig. 2:**
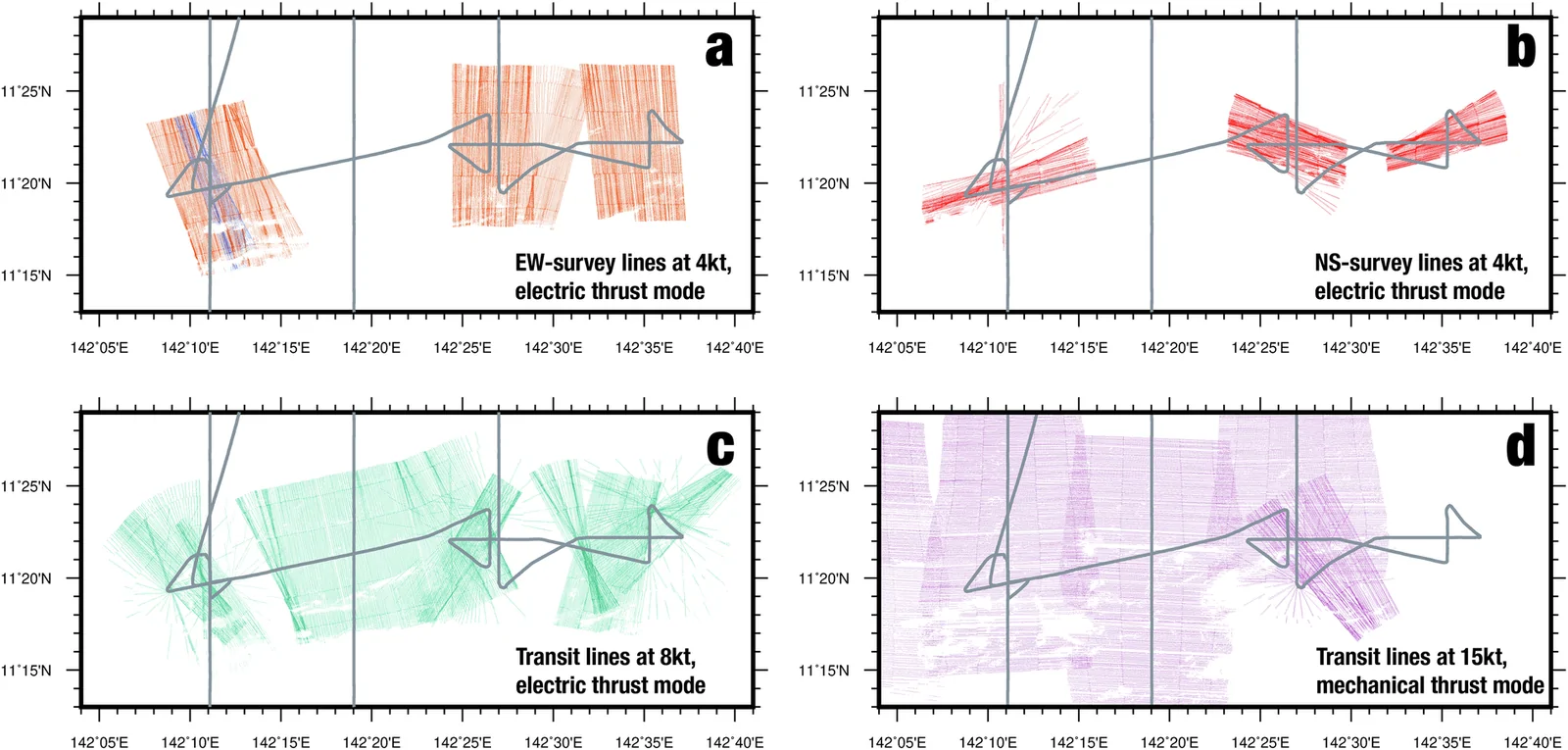
Data density of each sounding at various survey speeds and thrust modes in map view. Each panel shows sounding data distribution obtained along survey and transit lines; (**a**) east to west survey lines (orange) at 4 kt in electric thrust mode, (**b**) north to south survey lines at 4 kt in electric thrust mode, (**c**) transit lines at 8 kt in electric thrust mode, (**d**) transit lines at 15 kt in direct mechanical thrust mode. All surveys were conducted using EM124 sounding control of “Deeper Mode” except blue pings, in which “Very Deep Mode” was applied.

During the KH-23-9 cruise, east-west and north-south survey lines were planned across the eastern, central, and western basins of the Challenger Deep (Fig. 2). Observations were conducted at a vessel speed of 4 kt in electric-thrust mode, which provides a quieter and lower-vibration environment than mechanical propulsion, while the ship traveled between survey lines at 8 kt, and on some transits data were also collected at 15 kt under mechanical-thrust mode (Fig. 2, Table 1). For bathymetric acquisition, the Kongsberg EM124 multibeam echosounder was operated using predefined sonar settings optimized for full-ocean-depth measurements. In this study, the “Deeper” and “Very Deep” settings were used. These settings differ primarily in transmit pulse length, source level, and beam spacing. The “Very Deep” setting employs longer pulses and higher source levels to improve signal-to-noise ratio and bottom-detection capability at extreme depths. The longer pulse length may reduce range, or depth, resolution, whereas along-track sampling is controlled primarily by ping rate, beam geometry, and vessel speed. A 40°/40° swath angle was applied for the main survey, and the “Very deep” setting was operationally tested near the deepest area.

**Table 1:** Data list of the Challenger Deep sounded during the KH-23-9 cruise.

File name	Date	Location	Direction	Speed	Thrust mode
**4 kt mode using electric thruster**					
0188_20231130_023928.kmall	20231130	Eastern basin	W–E	4 kt	Electric
0189_20231130_030928.kmall	20231130	Eastern basin	W–E	4 kt	Electric
0190_20231130_033928.kmall	20231130	Eastern basin	W–E	4 kt	Electric
0192_20231130_041959.kmall	20231130	Eastern basin	N–S	4 kt	Electric
0195_20231130_052651.kmall	20231130	Central basin	E–W	4 kt	Electric
0196_20231130_055651.kmall	20231130	Central basin	E–W	4 kt	Electric
0197_20231130_062651.kmall	20231130	Central basin	E–W	4 kt	Electric
0199_20231130_071313.kmall	20231130	Central basin	S–N	4 kt	Electric
0200_20231130_074313.kmall	20231130	Central basin	S–N	4 kt	Electric
0204_20231130_092412.kmall	20231130	Western basin	E–W	4 kt	Electric
0205_20231130_095412.kmall	20231130	Western basin	E–W	4 kt	Electric
0206_20231130_100823.kmall	20231130	Western basin	E–W	4 kt	Electric
0209_20231130_111020.kmall	20231130	Western basin	N–S	4 kt	Electric
0210_20231130_114020.kmall	20231130	Western basin	N–S	4 kt	Electric
**Transit**					
0187_20231130_021850.kmall	20231130	Transit	SW–NE	8 kt	Electric
0191_20231130_035235.kmall	20231130	Transit	SE–NW	8 kt	Electric
0194_20231130_045651.kmall	20231130	Transit	SE–NW	8 kt	Electric
0198_20231130_064403.kmall	20231130	Transit	NW–SE	8 kt	Electric
0201_20231130_075412.kmall	20231130	Transit	NE–SW	8 kt	Electric
0202_20231130_082412.kmall	20231130	Transit	NE–SW	8 kt	Electric
0203_20231130_085412.kmall	20231130	Transit	NE–SW	8 kt	Electric
0207_20231130_103823.kmall	20231130	Transit	SE–NW	8 kt	Electric
0208_20231130_104020.kmall	20231130	Transit	SE–NW	8 kt	Electric
0211_20231130_114722.kmall	20231130	Transit	SW–NE	8 kt	Electric
**15 kt mode using direct mechanical thrust**					
0151_20231129_083516.kmall	20231129	Western basin	S–N	15 kt	Mechanical
0152_20231129_090015.kmall	20231129	Western basin	S–N	15 kt	Mechanical
0164_20231129_150014.kmall	20231129	Western basin	N–S	15 kt	Mechanical
0165_20231129_153014.kmall	20231129	Western basin	N–S	15 kt	Mechanical
0171_20231129_183014.kmall	20231129	Central basin	S–N	15 kt	Mechanical
0172_20231129_190014.kmall	20231129	Central basin	S–N	15 kt	Mechanical
0184_20231130_010014.kmall	20231130	Eastern basin	N–S	15 kt	Mechanical
0185_20231130_013014.kmall	20231130	Eastern basin	N–S	15 kt	Mechanical
0186_20231130_020014.kmall	20231130	Eastern basin	N–S	15 kt	Mechanical
**“EM124 Very Deep Mode” at 4kt**					
0212_20231130_120610.kmall	20231130	Western basin	E–W	4 kt	Electric

An expendable conductivity, temperature, and depth probe (XCTD4; The Tsurumi-Seiki Co., LTD) measured the upper-ocean temperature and salinity structure down to 1,914 m. The profile was extended below this depth using an ocean model to generate a full sound velocity profile. In addition, three XCTD1 profiles to 1,085 m were obtained nearby. During acquisition, an artifact appeared intermittently in the outer swath beams—likely at the sector boundary—resulting in sporadically deeper values occurring roughly once every few pings. These anomalous soundings were addressed during the beam-editing and cleaning workflow described below.

### Seawater sound velocity correction

**Fig. 3:**
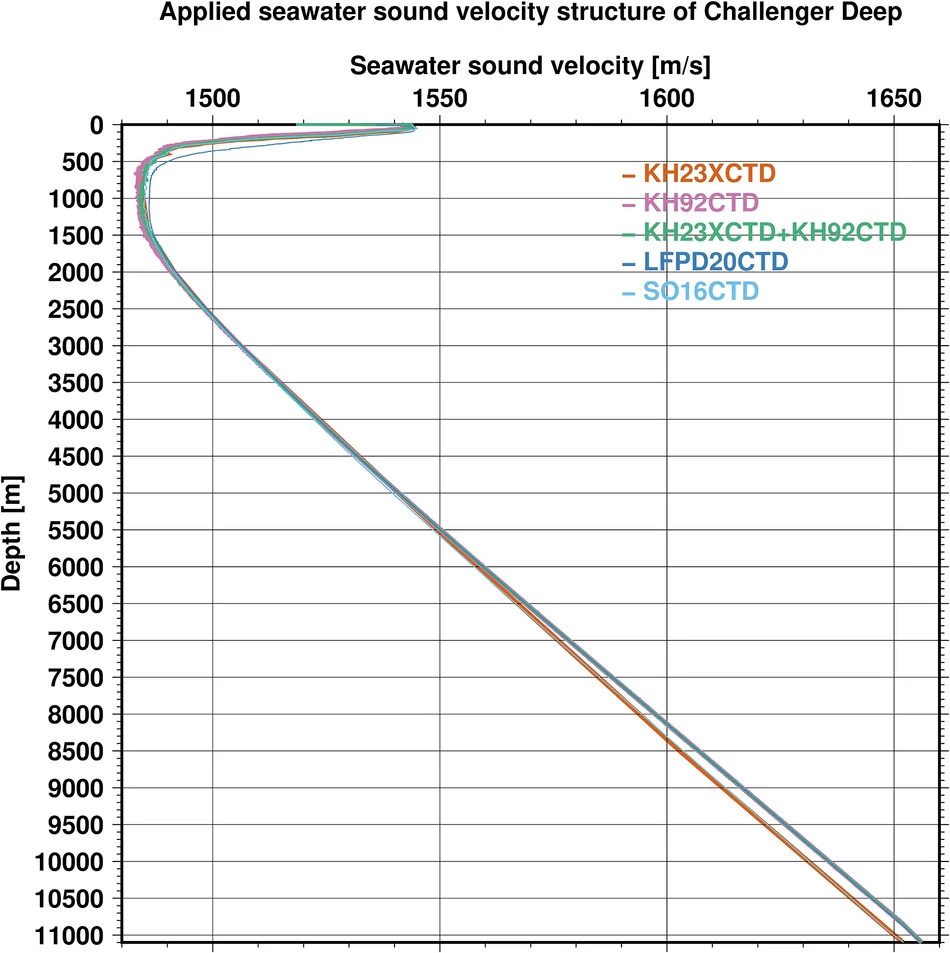
Applied profile of seawater sound velocity structure for sounding data correction. The applied seawater sound velocity models of KH23XCTD, KH92CTD, KH23XCTD+KH92CTD, LFPD20CTD, and SO16CTD are drawn with vermillion, purple, green, blue, and sky blue lines, respectively. Below 5,000 m depth, KH92CTD, KH23XCTD+KH92CTD, and LFPD20CTD are nearly overlapping, whereas KH23XCTD and SO16CTD are broadly similar and show slightly lower sound velocities than the other profiles.

Five seawater sound velocity (SSV) models were prepared to compute alternative depth solutions and to test the sensitivity of the bathymetric estimates to water-column structure: KH23XCTD, KH92CTD, KH23XCTD+KH92CTD, LFPD20CTD, and SO16CTD (Fig. 3). Except for LFPD20CTD, sound velocity was calculated using the Del Grosso^12^ equation. The LFPD20CTD was computed using TEOS-10, although resulting sound velocities are effectively identical to those calculated using the Del Grosso equation^12^.

The KH23XCTD model was generated onboard during the KH-23-9 expedition, using the XCTD profile obtained on 30 November 2023 (02:18 UTC), combined with climatological values from the Levitus dataset^13^. This model represents the sound velocity correction available during near-real-time shipboard processing.

The KH92CTD model was based on the full-depth CTD profile collected during R/V Hakuho-maru KH-92-1 cruise in 1992^5^. This profile provides a full-depth observational reference from the Challenger Deep region.

The KH23XCTD+KH92CTD model combined the KH-23-9 XCTD data from 0 to 1,915 m with the KH-92-1 full-depth CTD data below 1,915 meters. This model was used as the preferred SSV correction for bathymetric mapping in this study because it combines the contemporaneous upper-ocean structure measured during KH-23-9 with a full-depth CTD profile from the Challenger Deep region. The tested profiles differ most strongly in the upper ocean, although differences in the full-depth harmonic sound-speed structure also affect the final depth estimates.

The LFPD20CTD model is based on lander-based full-depth CTD profiles obtained by the DSV *Limiting Factor* during the DSSV *Pressure Drop* expedition in June 2020^10,11^. This campaign provided 18 profiles acquired between 7 and 26 June 2020, with maximum measured depths ranging from 10,725 m to 10,928 m. Because these profiles show some variation, especially in the layer shallower than 2,000 m, they were averaged using a Gaussian filter to generate the final model. In this study, LFPD20CTD was used as one of the independent full-depth reference models to evaluate the sensitivity of the KH-23-9 EM124 depths to the choice of SSV structure.

The SO16CTD model was calculated from the full-depth CTD profile obtained during the R/V *Sonne* expedition, using the corrected conservative temperature and absolute salinity values described by van Haren *et al*.^9,14^. Sound velocity was calculated using the Del Grosso^12^ equation for consistency with the KH23XCTD, KH92CTD, and KH23XCTD+KH92CTD models. The resulting SO16CTD sound velocity profile is broadly similar to the KH23XCTD model. The inclusion of SO16CTD therefore allows us to evaluate whether the KH-23-9 depth estimates are sensitive to the use of this independent full-depth profile.

### Data processing

**Fig. 4:**
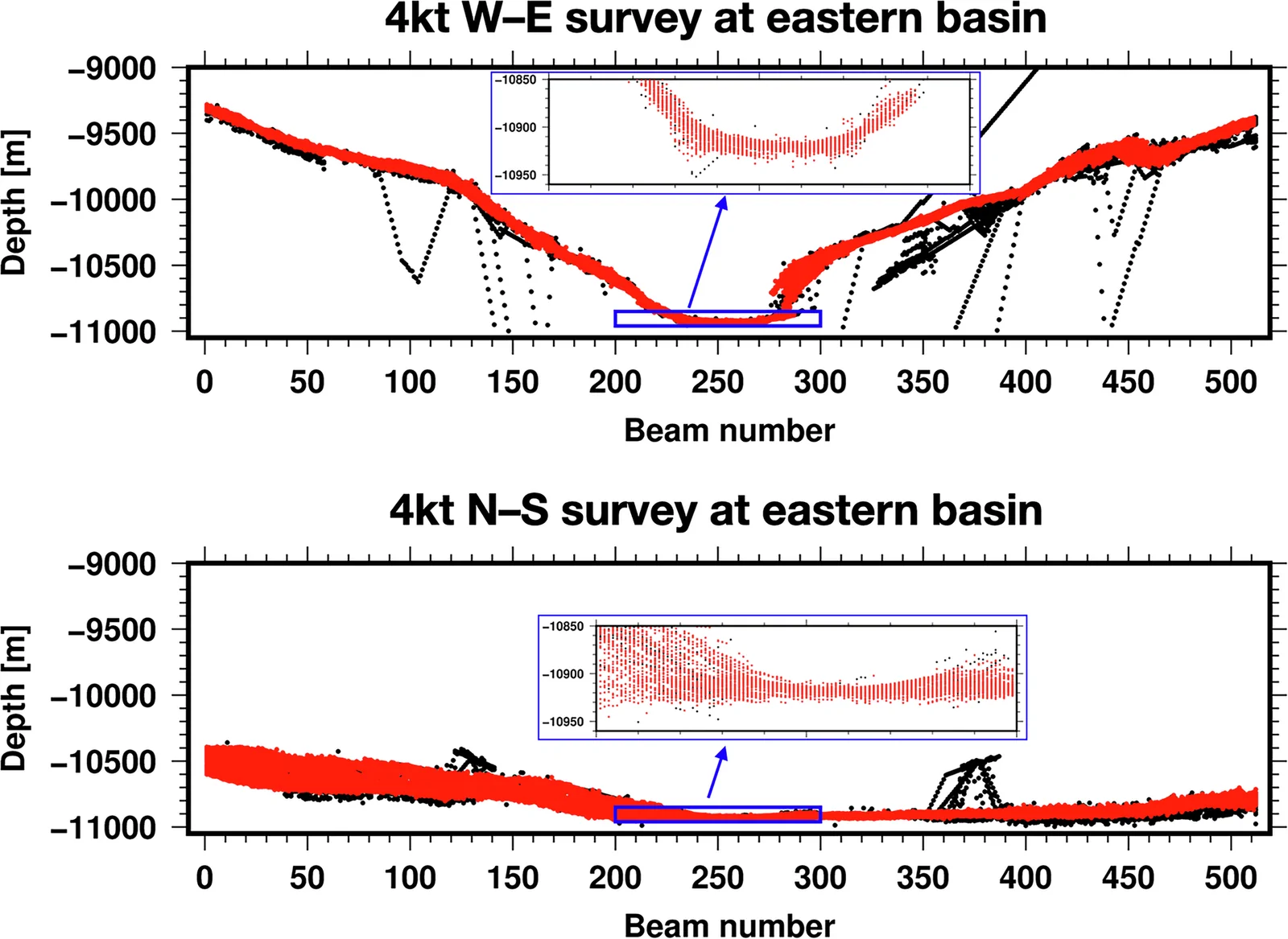
Swath data of each echo sounding at the deepest area of the eastern basin collected at ship speed of 4 kt along west to east (top) and north to south (bottom) survey lines. Red circles indicate accepted soundings, and black circles indicate rejected soundings.

Bathymetry was derived for each beam footprint (e.g., Fig. 4). The raw .kmall files were imported into HIPS and SIPS software (CARIS, Ltd., Fredericton, Canada), where vessel configuration parameters, navigation, attitude, heading, and the selected sound velocity profile were applied. Soundings were recomputed for each SSV model described above. To remove spurious depth excursions caused by acoustic noise, sector-boundary artifacts, and complex ray-path propagation, each ping was manually inspected using the swath and subset editing tools in CARIS. Rejected soundings were mainly isolated spikes or anomalously deep values occurring in the outer parts of the multibeam swath. No automated uncertainty-based filtering was applied; instead, the acceptance or rejection status of each sounding was retained in the exported point dataset.

To characterize sounding retention across survey modes, we calculated acceptance and rejection statistics for beams 1–512 in the edited sounding tables (Table 2). Because beam-angle information was not included in the exported point dataset, beams were grouped by their across-swath beam number into central beams, mid-swath beams, and outer-swath beams. The EW 4 kt electric-thrust survey lines had the lowest overall rejection rate of 13.0%, whereas the NS 4 kt electric-thrust lines and the 15 kt mechanical-thrust transit lines had higher rejection rates of 20.1% and 22.2%, respectively. The 8 kt electric-thrust transit lines had an intermediate rejection rate of 15.6%. In the EW 4 kt lines, the rejection rate increased from 6.7% in the central beams to 19.8% in the outer swath. A similar central-to-outer increase was observed in the 8 kt electric-thrust transit data, from 12.0% to 22.1%. In contrast, the NS 4 kt lines showed relatively high rejection rates across the swath, consistent with the greater difficulty of mapping slope-parallel morphology along the north–south lines. The 15 kt mechanical-thrust transit data retained useful depth information but had the highest overall rejection rate and the highest outer-swath rejection rate, supporting their use as supplementary rather than primary bathymetric constraints.

**Table 2:** Sounding rejection rates by survey mode and across-swath beam group.

Survey mode	Across-swath beam group	Reject rate
EW 4 kt electric	Outer-swath beams	19.8%
	Mid-swath beams	12.6%
	Central beams	6.7%
NS 4 kt electric	Outer-swath beams	19.7%
	Mid-swath beams	19.1%
	Central beams	21.7%
Transit 8 kt electric	Outer-swath beams	22.1%
	Mid-swath beams	13.3%
	Central beams	12.0%
Transit 15 kt mechanical	Outer-swath beams	27.3%
	Mid-swath beams	20.7%
	Central beams	19.0%

Rejection rates were calculated from the edited sounding tables after manual swath editing in CARIS. Beams 1–512 were grouped by across-swath beam number into outer-swath beams (1–77 and 436–512), mid-swath beams (78–179 and 334–435), and central beams (180–333). Percentages indicate the number of rejected soundings divided by the total number of soundings in each survey-mode and beam-group category. EW and NS denote east–west and north–south survey lines, respectively.

After ping-by-ping cleaning, depth values from accepted beams were exported as point clouds. These data were gridded using the GMT software’s “surface” algorithm, which applies an adjustable-tension continuous-curvature spline. GMT was used for final grid generation to apply the same reproducible gridding parameters to the point clouds produced with each SSV model. Grid spacings of 50, 100, 200, and 500 m were tested, with a search radius set to 1.5 times the grid spacing, and the resulting maximum gridded depths are summarized in Table 3. The grid-spacing test showed that the identification of the three >10,900 m basins was stable across the tested grid spacings, whereas single-cell maximum depths varied at the meter scale. The 50 m grid produced unstable single-cell extrema and was therefore used only as a gridding-sensitivity check, not for reporting the preferred depth range. A 100 m grid spacing was ultimately adopted to balance smoothness, spatial detail, and data coverage. The edited sounding cloud is locally denser than the adopted 100 m grid spacing, particularly along the central portions of the swaths, where accepted soundings are commonly spaced at several tens of meters to approximately 100 m. However, this high sounding density should not be interpreted as equivalent to the effective acoustic resolution. At a water depth of approximately 10,920 m, the nadir footprint width of a 2° EM124 beam is on the order of 400 m under a simple flat-seafloor geometric approximation. The footprint increases away from nadir because of oblique beam geometry and may also vary with seafloor slope. Thus, neighbouring soundings are not fully independent at the 100 m scale. The 100 m grid was used as a common interpolation grid for comparing depth solutions among sound velocity models, rather than to imply 100 m-scale acoustic resolving power.

**Table 3:** Sensitivity of maximum gridded depths to sound velocity model and grid spacing.

Seawater sound velocity model	Maximum depth			
	50 m	100 m	200 m	500 m
KH23XCTD	11021	10916	10913	10911
KH92CTD	11030	10925	10922	10919
KH23XCTD+KH92CTD	11029	10927	10924	10921
LFPD20CTD	10940	10932	10929	10926
SO16CTD	11018	10914	10911	10909

Maximum depths were calculated from gridded bathymetric surfaces generated from the edited sounding cloud using the same GMT “surface” workflow. The KH23XCTD+KH92CTD model is the preferred sound velocity correction used for the main bathymetric grid in this study. Alternative sound velocity models and grid spacings were tested to evaluate the sensitivity of single-cell maximum gridded depths to water-column structure and interpolation choices. The values should therefore be interpreted as gridded depth estimates for each processing case, rather than independent determinations of the absolute deepest point.

### Vertical reference frame

The horizontal coordinates of the bathymetric data are referenced to WGS-84. The depths presented in this dataset are sea-surface-referenced acoustic depths derived from the standard EM124/SIS and CARIS processing workflow, rather than ellipsoidally referenced depths. Vessel installation parameters, including the transducer draft, position offsets, attitude offsets, and motion-sensor configuration, were applied through the vessel configuration used during acquisition and processing. No independent ellipsoidally referenced survey workflow was applied, and no geoid, mean sea surface, or chart-datum separation model was used to reduce the bathymetry to an external vertical datum.

No external tidal or water-level correction was applied to the final gridded bathymetric surfaces. The Challenger Deep is far from a nearby tide gauge, and the expected water-level variability is small compared with the dominant sound-velocity-related sensitivity examined in this study. Nevertheless, residual water-level variability and dynamic draft are components of the vertical uncertainty budget and are considered as unresolved vertical-reference terms in the uncertainty assessment below. Therefore, the reported maximum depths should be interpreted as gridded EM124 acoustic depths referenced to the survey sea surface, with meter-scale comparisons to pressure-derived or externally datum-referenced depths requiring caution.

## Data Records

Two related datasets are deposited in the Arctic and Antarctic Data archive System (ADS). The seawater sound velocity model dataset is available at https://doi.org/10.17592/001.2026032302^15^, and the bathymetric dataset is available at https://doi.org/10.17592/001.2026032303^16^. The contents, formats, field definitions, and naming conventions are described below.

### Seawater sound velocity model dataset

The seawater sound velocity model dataset contains five ASCII profile files:

- KH23XCTD.asvp, KH-23-9 XCTD profile extended to full ocean depth.
- KH23XCTD+KH92CTD.svp, preferred composite profile combining the KH-23-9 XCTD and KH-92-1 full-depth CTD.
- KH92CTD.svp, full-depth profile based on the KH-92-1 CTD.
- LFPD20CTD.svp, profile based on the 2020 DSV Limiting Factor full-depth CTD observations.
- SO16CTD.svp, profile based on the 2016 R/V Sonne full-depth CTD.

In KH23XCTD.asvp, a Kongsberg ASVP header is followed by two whitespace-delimited numeric columns: depth (m) and sound speed (m s−1). The four.svp files begin with [SVP_VERSION_2], followed by a source/path record, a Section record containing the profile date/time and position, and the same depth and sound-speed columns. The source/path record documents provenance and is not a numeric data field. Each profile extends to 12,000 m using measured and/or extended values as described in Methods.

### Bathymetric dataset

The bathymetric dataset has the following top-level structure:

- mbes_vesselconfig/HAKUHO_EM124_20220615.hvf, CARIS HIPS vessel-configuration file.
- mbes_griddata/, 20 gridded bathymetric surfaces in GMT netCDF-4.grd format.
- mbes_kmalldata.zip, compressed raw Kongsberg EM124.kmall files.
- mbes_xyzdata.zip, compressed, processed sounding point files exported after recomputation and manual editing in CARIS.
- prc_makegrid.sh, shell script used to generate the released grids from the processed point files.

The .hvf file is an XML HIPS Vessel Configuration (version 2) file containing the vessel shape and the navigation, attitude, sound-velocity, depth-sensor, waterline, and transducer offset and mounting parameters used for processing. The raw archive preserves the native Kongsberg datagrams; the vessel configuration is supplied so that the acquisition geometry and applied offsets can be inspected.

The processed sounding files in mbes_xyzdata.zip are comma-delimited text. The fields used by prc_makegrid.sh are column 1, latitude (decimal degrees north); column 2, longitude (decimal degrees east); column 3, positive-down acoustic depth (m); and column 10, the CARIS edit-status string (Accept or Reject). The intervening columns retain ancillary CARIS-export metadata and are not used by the released gridding script.

Files in mbes_griddata/ follow the pattern bathy_KH-23-9-cdeep-4ktEW+8kt-<SSV-tag>_surf-I<grid-spacing>m-S<mask-radius>m.grd. The five SSV tags are caladan_sv_lander_clean_avg_n18, KH-23-9_11n143e_xctd_nov30, svp-DG_CTD-Hakuho92, svp-DG_SO16CTD_20260727, and svp-DG_XCTD-Hakuho23+CTD-Hakuho92; these correspond to LFPD20CTD, KH23XCTD, KH92CTD, SO16CTD, and KH23XCTD+KH92CTD, respectively. Each tag is provided at four grid-spacing/mask-radius combinations: 50/75 m, 100/150 m, 200/300 m, and 500/500 m, giving 20 files in total.

Each .grd file is a GMT netCDF-4 grid whose metadata declare the COARDS and CF-1.5 conventions. It contains coordinate variables lon (degrees east) and lat (degrees north), and a two-dimensional z field in metres. Horizontal positions are WGS-84. The script converts positive-down sounding depths to negative z values below the survey sea surface; unsupported cells outside the specified data mask are stored as missing values. The grid region is 141–144°E and 10–13°N.

The released script reads only soundings marked Accept, applies GMT blockmedian and surface with tension 0.65, constructs a data-support mask with grdmask, and sets cells outside that mask to missing. The netCDF histories identify GMT 5.4.5 as the software version used for the archived grids.

## Technical Validation

**Fig. 5:**
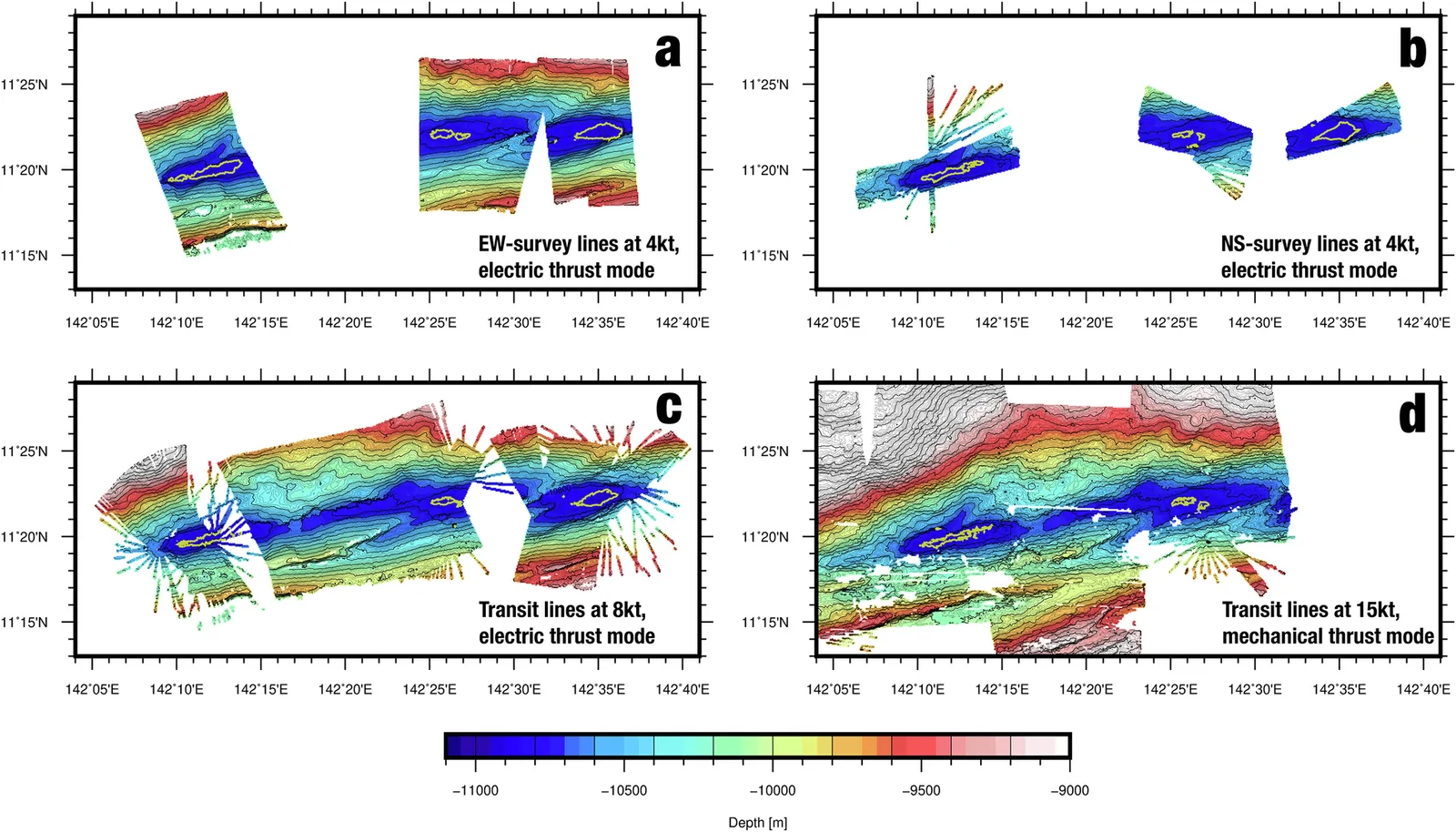
Bathymetric maps obtained by each sounding at various survey speeds and modes. Each panel shows depth data distribution obtained along survey and transit lines; (**a**) east to west survey lines at 4 kt in electric thrust mode, (**b**) north to south survey lines at 4 kt in electric thrust mode, (**c**) transit lines at 8 kt in electric thrust mode, (**d**) transit lines at 15 kt in direct mechanical thrust mode. The contour interval is 20 m. The yellow contour shows water depth of 10,900 m.

On the east-west survey lines, the 4 kt electric-thrust mode provided a high density of accepted soundings, producing smooth gridded bathymetric surfaces (Fig. 5a). Localized data gaps occurred in acoustic shadow zones near ridge structures. Along the north–south lines, the central beams (within ~1 km of the ship track) captured the basin floor consistently, whereas the outer swaths were noisier, which may partly reflect vessel pitch and roll induced by currents and wind. Strong along-track contour variations were also observed on the north–south slopes (Fig. 5b), indicating that slopes aligned with the vessel’s heading are more difficult to represent accurately in the gridded surface.

Data collected at 8 kt under electric thrust during transits between basins yielded terrain grids of comparable quality to those at 4 kt (Fig. 5c). At 15 kt in mechanical-thrust mode, the reduced numbers of pings resulted in sparser coverage, although depths exceeding 10,920 m were still detected in the eastern basin (Fig. 5d). Based on these assessments, the east–west lines acquired at 4 and 8 kt provided the most suitable coverage for constructing the preferred bathymetric grid.

**Fig. 6:**
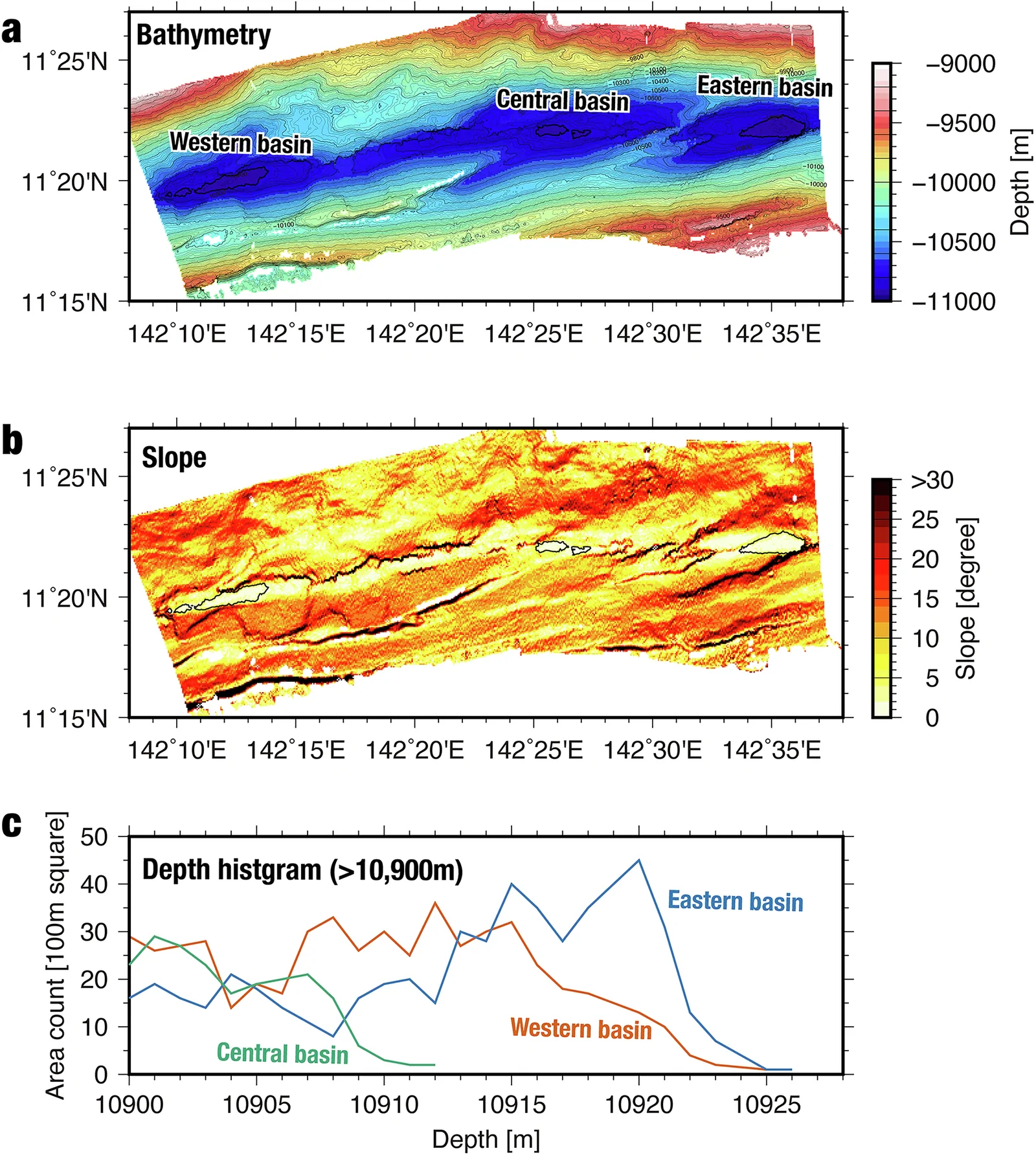
Bathymetric map (**a**), slope map (**b**), and depth histograms for areas deeper than 10,900 m in three basins (**c**). The contour interval is 20 m. The thick contour shows a depth of 10,900 m.

The preferred gridded bathymetry delineates three depressions in the Challenger Deep, each exceeding 10,900 m in depth (Fig. 6a). These basins are bounded by steep, north- and south-facing slopes (Fig. 6b), and separated by 200–300-m-high topographic highs, consistent with the segmented morphology of the Pacific Plate in this region. In the preferred KH23XCTD+KH92CTD solution, the maximum gridded depths of the western, central, and eastern basins are 10,926 m, 10,912 m, and 10,927 m, respectively. Their areas deeper than 10,900 m are 5.28, 2.00, and 5.33 km^2^ (Fig. 6c). The eastern basin contains an extensive area of seafloor near 10,920 m in this gridded solution. These values describe the preferred gridded processing case and are not independent absolute-depth determinations.

The overall shapes of the three basins are consistent with previous findings^7,11^. In the preferred KH23XCTD+KH92CTD solution, the maximum gridded EM124 depth in the eastern basin is 10,927 m. This value is shallower than the pressure-derived depth of 10,935 m ± 6 m reported by Greenaway *et al*.^11^, although the LFPD20CTD-based solution from the present dataset gives a deeper maximum gridded depth of 10,932 m. These comparisons indicate that the basin-scale morphology is reproducible among modern surveys, whereas single-cell maximum depths remain sensitive to sound velocity structure, vertical referencing, beam footprint, and gridding choices.

### Uncertainty framework

Guided by the general structure of multibeam error-budget and bathymetric-uncertainty approaches developed for hydrographic surveys^17,18^, and by recent TPU-based approaches for interpreting repeated multibeam bathymetric surveys^19^, we identify the principal sources of vertical uncertainty relevant to the KH-23-9 EM124 dataset.

The purpose of this study is to release and validate a full-ocean-depth EM124 bathymetric dataset and to evaluate the sensitivity of the resulting gridded depths to key survey and processing parameters. The survey was not designed as an ellipsoidally referenced hydrographic survey, and we do not derive a statistically independent 95% confidence interval for the deepest point. Instead, following the general structure of multibeam uncertainty-budget approaches developed for hydrographic surveys, we identify the principal sources of vertical uncertainty and separate them into components that can be evaluated directly from the present dataset and components that remain as residual or unresolved terms.

The largest directly testable contribution in this study is the seawater sound velocity structure. Reprocessing the same edited sounding dataset with five SSV models yields maximum gridded depths from 10,914 to 10,932 m, with the preferred KH23XCTD+KH92CTD solution giving 10,927 m. This range should be interpreted as a sensitivity envelope associated with the applied sound velocity models, not as a formal total propagated uncertainty or total vertical uncertainty surface. Other factors, including water-level variability, dynamic draft, vessel-motion residuals, beam footprint, sounding density, editing choices, and gridding, also contribute to the uncertainty of single-cell maximum depths, but cannot be fully isolated from the present dataset alone.

Table 4 summarizes the indicative uncertainty and sensitivity scales relevant to the KH-23-9 EM124 dataset. The values should not be interpreted as a formal total propagated uncertainty or as a statistically independent 95% confidence interval. Rather, they provide an order-of-magnitude framework for comparing the dominant directly tested sensitivity, namely sound velocity model dependence, with additional residual terms related to vertical referencing, platform motion, beam geometry, editing, and gridding.

**Table 4:** Indicative uncertainty and sensitivity scales for the KH-23-9 EM124 bathymetric dataset.

Source	Indicative scale	Basis and interpretation
Sound velocity model dependence	−13 to +5 m relative to the preferred solution	Five SSV models applied to the same edited sounding dataset give maximum gridded depths of 10,914–10,932 m in the eastern basin. This is the largest directly quantified sensitivity in this study.
Preferred gridded solution	10,927 m	The KH23XCTD+KH92CTD model is used as the preferred solution because it combines the contemporaneous KH-23-9 upper-ocean XCTD profile with a full-depth CTD profile from the Challenger Deep region.
Upper-ocean sound velocity structure	~2 m	Replacing the upper 1,915 m of KH92CTD with the contemporaneous KH-23-9 XCTD profile changes the maximum gridded depth by approximately 2 m.
Full-depth reference profile choice	several to >10 m	Recent and historical full-depth reference models give different maximum gridded depths, including 10,932 m for LFPD20CTD and 10,914 m for SO16CTD.
Water level, vertical reference, and dynamic draft	likely sub-meter to meter scale	No external tidal, water-level, ellipsoid, or geoid/MSS (Mean Sea Surface) reduction was applied. These terms are treated as unresolved vertical-reference contributions and are expected to be smaller than the SSV-model sensitivity.
Vessel motion, attitude, and timing residuals	likely sub-meter to meter scale	Navigation, attitude, heading, and motion-sensor data were applied in the standard EM124/SIS and CARIS workflow. Residual effects are not independently quantified.
Outer-swath artifacts and editing	local meter-scale effect possible	Sector-boundary artifacts and anomalously deep outer-swath soundings were reduced by ping-by-ping manual editing. No formal swath-angle-dependent TVU (Total Vertical Uncertainty) surface was generated.
Beam-footprint geometry	meter-scale effect possible for local maxima	The 2° × 2° EM124 footprint may smooth or under-represent narrow depressions at full-ocean depth. This effect can influence single-cell maximum gridded depths but cannot be isolated from the present dataset alone.
Gridding and interpolation	meter-scale effect possible	Grid spacings of 50, 100, 200, and 500 m were tested. The adopted 100 m grid is an interpolation grid for comparing depth solutions and should not be interpreted as the effective acoustic resolution.
Pressure-to-depth gravity correction	not applicable to EM124 depths	EM124 depths are derived from acoustic travel time and sound velocity structure. Gravity corrections are relevant only when comparing with pressure-derived depths from previous studies.

To place the present results in the context of previous modern surveys, Table 5 summarizes recent Challenger Deep depth estimates and their uncertainty treatments. Compared with the formal or reported uncertainty estimates of Gardner *et al*.^8^, van Haren *et al*.^9^, Bongiovanni *et al*.^10^, and Greenaway *et al*.^11^, the present 10,927 m value should be interpreted as the preferred KH23XCTD + KH92CTD gridded EM124 solution, while the 10,914–10,932 m range represents a sound velocity model sensitivity envelope rather than a formal 95% confidence interval.

**Table 5:** Comparison of recent Challenger Deep depth estimates and uncertainty treatments.

Study	Platform	Instrument	Vertical / SSV control	Deepest value	Uncertainty
Gardner *et al*.^8^	USNS *Sumner*	EM122, 1° × 1°	XBT-based SSV, calibrated/checked against CTD	10,984 m	±25 m, 95%
van Haren *et al*.^9^	R/V *Sonne*	EM122, 0.5° × 1°	local full-ocean-depth CTD	10,925 m	±12 m
Bongiovanni *et al*.^10^	DSSV *Pressure Drop*	EM124, 1° × 2°	full-ocean-depth DSV/lander CTD	10,924 m	±15 m, 95%
Greenaway *et al*.^11^	DSV *Limiting Factor*	pressure-derived depth + acoustic altimeter transects	pressure-derived depth corrected for density structure, atmospheric pressure, water level, gravity anomaly, and vertical gravity gradient	10,935 m	±6 m, 95%
This study	R/V *Hakuho-maru*	EM124, 2° × 2°	five SSV sensitivity tests including full-ocean-depth CTDs	10,927 m preferred	10,914–10,932 m SSV sensitivity envelope

Reported deepest values, survey platforms, measurement methods, beamwidths, depth-control approaches, and uncertainty estimates are summarized for recent multibeam and pressure-derived measurements of the Challenger Deep. Beamwidths are listed where reported for shipborne multibeam systems; they are not applicable to the pressure-derived estimate of Greenaway *et al*.^11^. For this study, 10,927 m represents the preferred KH23XCTD+KH92CTD gridded EM124 solution, whereas 10,914–10,932 m indicates the sensitivity envelope obtained from five sound velocity models, not a formal 95% confidence interval.

### Effects of sound velocity structure

To evaluate the sensitivity of the gridded depths to sound velocity structure, we applied the five SSV models described above (Table 3). With the preferred KH23XCTD + KH92CTD model, the maximum gridded depth was 10,927 m, whereas the LFPD20CTD model yielded 10,932 m. The original CTD data for KH92CTD and LFPD20CTD were obtained more than 20 years apart and in different seasons, suggesting that temporal and seasonal differences in water-column structure can contribute to meter-scale differences in estimated depths. The SO16CTD model gives the shallowest maximum gridded depth of 10,914 m in the eastern basin, 13 m shallower than the preferred KH23XCTD + KH92CTD solution. This result further demonstrates that the choice of full-depth reference profile can shift single-cell maximum gridded depths by more than 10 m.

Applying the KH23XCTD and KH92CTD models alone gave maximum gridded depths of 10,916 m and 10,925 m, respectively. The 2 m difference between KH92CTD and KH23XCTD + KH92CTD reflects the effect of replacing the upper 1,915 m of the 1992 CTD profile with the contemporaneous KH-23-9 XCTD profile (Fig. 3). In contrast, the KH23XCTD-only solution gives a substantially shallower maximum depth, mainly because the onboard XCTD profile measured only the upper ocean and was extended below 1,914 m using climatological values. These results show that the upper 2,000 m of the water column is an important contributor to the sound velocity dependence of full-ocean-depth EM124 estimates, while also demonstrating the need to combine contemporaneous shallow observations with a full-depth CTD reference profile.

### Effects of vessel speed and operating mode

Data collected at 4 kt and 8 kt under electric-thrust mode produced the smoothest gridded bathymetric surfaces, reflecting the higher sounding density and quieter operating conditions. At 15 kt in mechanical-thrust mode, the reduced number of pings resulted in sparser coverage and a less continuous gridded surface. Nevertheless, depths exceeding 10,920 m were still detected in the eastern basin when an appropriate SSV model was applied. These results indicate that transit-speed EM124 data can provide useful supplementary constraints on basin-scale bathymetry in the deepest ocean, but they should not be regarded as equivalent in quality or spatial completeness to dedicated low-speed survey lines.

### Effects of EM124 beam-footprint geometry

A key instrumental factor in full-ocean-depth multibeam mapping is the acoustic beam footprint. The EM124 installed on the DSSV Pressure Drop was configured with a 1° × 2° transmit–receive beam footprint for the bathymetric survey reported by Bongiovanni *et al*.^10^, whereas the EM124 on R/V Hakuho-maru uses a 2° × 2° footprint. Although both systems share the same model number, EM124 installations allow multiple footprint configurations depending on transducer size and vessel integration.

A wider footprint insonifies a larger area of the seafloor and tends to average the returned signal. It can therefore smooth or under-represent narrow depressions and sharp topographic variations relative to a narrower-footprint system operating under otherwise similar conditions. This effect is particularly relevant in the Challenger Deep, where the local maximum depths may occur within small depressions or rough seafloor at the basin floors. The 2° × 2° footprint of the Hakuho-maru EM124 may therefore contribute to meter-scale differences in single-cell maximum gridded depths, especially when compared with narrower-footprint shipborne EM124 surveys or near-bottom observations.

However, the magnitude of the footprint effect cannot be isolated from the present dataset alone. Differences in sound velocity structure, vertical referencing, sounding density, editing decisions, and gridding choices also affect the deepest gridded values. We therefore interpret beam-footprint geometry as a plausible contributing factor, rather than as a sole explanation for differences between the present preferred gridded depth and previously reported deepest values. The robust result from the present comparison is that the basin-scale morphology is consistent among modern surveys, whereas the deepest local values remain sensitive to survey configuration and processing choices. Future cross-calibration with narrower-footprint shipborne systems or near-bottom high-resolution mapping would help quantify the footprint contribution more directly.

### Gravity correction in pressure-derived depth estimates

Pressure-derived depths require conversion of hydrostatic pressure to depth using local, depth-dependent gravity, together with atmospheric-pressure and water-level corrections. Greenaway *et al*.^11^ explicitly included regional gravity-anomaly and vertical gravity-gradient terms and showed that these corrections amount to approximately 4.2 m in the Challenger Deep area, although the total uncertainty remains dominated by pressure-sensor precision. By contrast, Taira *et al*.^5^ reported CTD pressure-derived depths using then-standard practice but did not report such site-specific gravity-anomaly or gravity-gradient corrections. The EM124 depths presented here are derived from two-way acoustic travel time and sound velocity structure, rather than from hydrostatic pressure, and therefore gravity correction is not a direct term in the MBES depth computation. Nevertheless, comparisons between pressure-derived and acoustic bathymetric depths should recognize that meter-scale differences may arise not only from sound velocity structure, beam-footprint geometry, vertical referencing, and gridding choices, but also from whether local gravity corrections were included in pressure-to-depth conversion^5,11^.

## Usage Notes

The raw .kmall files require software compatible with Kongsberg KMALL datagrams. The processed point files retain both accepted and rejected soundings; select the records whose column-10 status is Accept to reproduce the released grids. Point-file depths are positive downward, whereas the GMT grids store seafloor elevation as negative z values. The 100 m grid is an interpolation framework for comparing processing cases and should not be interpreted as 100 m acoustic resolution because the EM124 beam footprint is substantially wider at full-ocean depth. All reported depths are referenced to the survey sea surface, and no external tidal, geoid, mean-sea-surface, or chart-datum correction has been applied.

## Data Availability

The seawater sound velocity model dataset is archived in ADS and is available at https://doi.org/10.17592/001.2026032302^15^. The bathymetric dataset is archived separately in ADS and is available at https://doi.org/10.17592/001.2026032303^16^.

## Code availability

The shell script prc_makegrid.sh is distributed with the bathymetric dataset at https://doi.org/10.17592/001.2026032303^16^. It requires a POSIX-compatible shell, awk, and GMT 5.4.5. No other custom code is required to use the released files.

## References

[1] Carruthers, J. N. & Lawford, A. L. The deepest oceanic sounding. *Nature* **169**, 601–603 10.1038/169601a0 (1952).

[2] Piccard, J. & Dietz, R. S. Seven Miles Down: The Story of the Bathyscaph Trieste (G. P. Putnam’s Sons, 1961).

[3] Hydrographic Department, Japan Marine Safety Agency. Mariana Trench survey by the Takuyo. International Hydrographic Bulletin, September, 351–352 (1984).

[4] Fujimoto, H., Fujiwara, T., Kong, L. & Igarashi, C. Sea-Beam survey over the Challenger Deep, revisited. In Preliminary Report of the Hakuho-Maru Cruise KH-92-1, 26–27 (Ocean Research Institute, The University of Tokyo, 1993).

[5] Taira, K., Yanagimoto, D. & Kitagawa, S. Deep CTD casts in the Challenger Deep, Mariana Trench. *Journal of Oceanography* **61**, 447–454 10.1007/s10872-005-0053-z (2005).

[6] Fujioka, K., Okino, K. & Kanamatsu, T. Morphology and origin of the Challenger Deep in the southern Mariana Trench. *Geophysical Research Letters* **29**, 1372 10.1029/2001GL013595 (2002).

[7] Nakanishi, M. & Hashimoto, J. A precise bathymetric map of the world’s deepest seafloor, Challenger Deep in the Mariana Trench. *Marine Geophysical Research* **32**, 455–463 10.1007/s11001-011-9134-0 (2011).

[8] Gardner, J. V., Armstrong, A. A., Calder, B. R. & Beaudoin, J. So, how deep is the Mariana Trench? *Marine Geodesy* **37**, 1–13 10.1080/01490419.2013.837849 (2014).

[9] van Haren, H., Berndt, C. & Klaucke, I. Ocean mixing in deep-sea trenches: new insights from the Challenger Deep, Mariana Trench. *Deep-Sea Research Part I* **129**, 1–9 10.1016/j.dsr.2017.09.003 (2017).

[10] Bongiovanni, C., Stewart, H. A. & Jamieson, A. J. High-resolution multibeam sonar bathymetry of the deepest place in each ocean. *Geoscience Data Journal* **9**, 108–123 10.1002/gdj3.122 (2021).

[11] Greenaway, S. F., Sullivan, K. D., Umfress, S. H., Beittel, A. B. & Wagner, K. D. Revised depth of the Challenger Deep from submersible transects; including a general method for precise, pressure-derived depths in the ocean. *Deep-Sea Research Part I* **178**, 103644 10.1016/j.dsr.2021.103644 (2021).

[12] Del Grosso, V. A. New equation for the speed of sound in natural waters (with comparisons to other equations). *Journal of the Acoustical Society of America* **56**, 1084–1091 10.1121/1.1903388 (1974).

[13] Levitus, S. Climatological Atlas of the World Ocean. NOAA Professional Paper 13 (U.S. Government Printing Office, 1982).

[14] van Haren, H., Uchida, H. & Yanagimoto, D. Further correcting pressure effects on SBE911 CTD-conductivity data from hadal depths. *Journal of Oceanography* **77**, 137–144 10.1007/s10872-020-00565-3 (2021).

[15] Fujii, M. & Tamura, C. Seawater sound velocity model dataset for full-ocean-depth EM124 mapping of the Challenger Deep by R/V Hakuho-maru. Version 1.00. *Arctic and Antarctic Data archive System (ADS)*, 10.17592/001.2026032302 (2026).42749747

[16] Fujii, M. & Tamura, C. Bathymetric dataset for full-ocean-depth EM124 mapping of the Challenger Deep by R/V Hakuho-maru. Version 1.00. Arctic and Antarctic Data archive System (ADS) 10.17592/001.2026032303 (2026).42749747

[17] Hare, R. Depth and position error budgets for multibeam echosounding. *International Hydrographic Review* **72**, 37–69 (1995).

[18] Hare, R., Eakins, B. W. & Amante, C. Modelling bathymetric uncertainty. *International Hydrographic Review* **6**, 31–42 (2011).

[19] Sauter, G., Fabbri, S. C., Frischknecht, C., Anselmetti, F. S. & Kremer, K. A systemic approach to managing uncertainties in repetitive multibeam bathymetric surveys. *Environmental Modelling & Software* **186**, 106333 10.1016/j.envsoft.2025.106333 (2025).

